# Mini-thoracotomy and full-sternotomy approach for reoperative mitral valve surgery after a previous sternotomy

**DOI:** 10.1093/icvts/ivab309

**Published:** 2021-11-22

**Authors:** Yelee Kwon, Sung Jun Park, Ho Jin Kim, Joon Bum Kim, Sung-Ho Jung, Suk Jung Choo, Jae Won Lee

**Affiliations:** Department of Thoracic and Cardiovascular Surgery, Asan Medical Center, University of Ulsan College of Medicine, Seoul, South Korea

**Keywords:** Mini-thoracotomy, Reoperative, Mitral valve

## Abstract

**OBJECTIVES:**

Right mini-thoracotomy approach may enhance the visualization of mitral valve (MV) visualization during redo MV surgery, thereby minimizing the risk of reoperative median sternotomy. We described the clinical outcomes of redo MV surgery by mini-thoracotomy and full-sternotomy approach.

**METHODS:**

Of 730 consecutive adult patients who underwent redo MV surgery between 2002 and 2018 at our institution, we identified 380 patients (age: 56.0 [14.8] years) after excluding those who underwent concomitant aortic valve or coronary artery surgeries.

**RESULTS:**

The clinical outcomes in patients who underwent mini-thoracotomy (MINI group; *n* = 168) and full-sternotomy (STERN group; *n* = 218) were described. The early and overall mortality in the MINI group was 4.3% (7/162) and 17.3% (28/162), with the rates of early major complications as follows: low cardiac output syndrome, 5.6% (9/162); early stroke, 6.8% (11/162); new-onset dialysis, 6.2% (10/162); prolonged ventilation, 15.4% (25/162); and postoperative bleeding requiring exploration, 7.4% (12/162). In the STERN group, the early mortality was 11.0% (24/218), whereas the risk of low cardiac output syndrome, early stroke, new-onset dialysis, prolonged ventilation, and postoperative bleeding was 12.4% (27/218), 14.2% (31/218), 17.0% (37/218), 33.0% (72/218), and 10.1% (22/218), respectively. The duration of intensive care unit and hospital stay was 2.0 [range 1.0, 3.0] and 8.0 [6.0, 13.0], respectively, in the MINI group and 3.0 [2.0, 7.0] and 14.0 [8.0, 29.0], respectively, in the STERN group.

**CONCLUSIONS:**

Mini-thoracotomy may be a viable alternative to conventional sternotomy for redo MV surgery.

**Figure:**
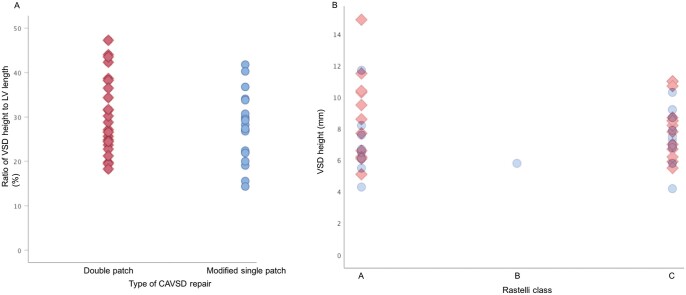

## INTRODUCTION

Redo cardiac surgery via median sternotomy is technically challenging due to the risk of damaging critical cardiac structures that are tightly adhered behind to the sternum, such as the ascending aorta, right ventricle and coronary bypass graft [1, 2]. Redo sternotomy may also be difficult in patients who had mediastinitis or sternal wound infections previously. With improving postoperative outcomes and prolonged life expectancies, the number of reoperative valve surgeries has also increased. According to Park *et al.* [3], 13.5% of all cardiac procedures at the Mayo clinic required repeat median sternotomies, 35% of which were mitral valve (MV) surgery.

Right mini-thoracotomy MV surgery may have excellent short- and long-term results that are comparable to a conventional sternotomy. During redo MV surgery, right mini-thoracotomy may help minimize the intraoperative risk by avoiding the damage to adjacent cardiac structures and offering enhanced visualization of the MV without extensive mediastinal dissection [4, 5]. Right thoracotomy for reoperative MV can be safely performed [4, 5]. However, limited information is available regarding the parallel description of outcomes in mini-thoracotomy and sternotomy approach. Therefore, we described the postoperative clinical outcomes of mini-thoracotomy and full sternotomy in patients who underwent redo MV surgery.

## PATIENTS AND METHODS

### Ethics statement

This study was approved by the Asan Medical Center Ethics Committee and Review Board on 13 December 2019 (No: 2019-1617), which waived the requirement for informed patient consent because of the retrospective nature and anonymity of the study.

### Patients

We included the patients who underwent reoperative MV surgery after a previous sternotomy at our institution between January 2002 and July 2018. Patients who underwent concomitant aortic valve (AV) or coronary bypass surgeries that mandated a sternotomy approach were excluded. All data were obtained from the medical records.

The primary outcomes of interest were early and overall mortality. Early mortality was defined as a death occurring within 30 days of surgery or during the same hospitalization after surgery. The secondary outcomes of interest were early major complications that included low cardiac output syndrome (LCOS) requiring mechanical circulatory support, in-hospital stroke, postoperative bleeding requiring exploration, new-onset dialysis, prolonged ventilation (>24 h) and the amount of blood transfusions in unit. For efficacy outcomes, recurrent mitral regurgitation (MR) was defined as those greater than moderate degree of MR, including paravalvular leakage on follow-up echocardiography or reoperation of MV because of MR.

Follow-up data were obtained until 01 July 2020. Patients without clinical events were censored at the end of the follow-up. Vital status was checked through the institutional medical records and the National Population Registry of the Korea National Statistical Office. All relevant data are within the manuscript and its Supplementary Material files.

### Surgical technique

Patients in the MINI group received a thoracoscopy-guided, right anterolateral 7–10-cm incision through the fourth intercostal space. The patients were placed in the left semi-lateral decubitus position with the right chest elevated at ∼30°. Arterial cannulation was performed using the femoral artery, and venous cannulation was performed via the femoral vein and right internal jugular vein for cardiopulmonary bypass (CPB). No patients underwent rib resection. The ascending aorta was cross-clamped with a transthoracic clamp, and anterograde cardioplegia was delivered into the aortic root, except in 9 patients in whom fibrillatory arrest was employed. Patients were generally systemically cooled to 28°C.

Patients in the STERN group underwent repeat median sternotomy in a standard fashion with an oscillating saw. All patients underwent preoperative heart computed tomography; if there was close proximity of the posterior sternal table and ascending aorta on the computed tomography, femoral CPB was initiated before sternotomy to mitigate the risk of aortic rupture during sternal re-entry, at the discretion of each surgeon.

### Statistical analysis

Statistical analysis was performed using R version 3.6.3 (R Foundation for Statistical Computing, Vienna, Austria; https://www.r-project.org/). Continuous variables were presented as mean (standard deviation) or median with interquartile range. Categorical variables were presented with proportions or frequencies. Between-group differences were compared using Student’s *t*-test for continuous variables and Pearson’s chi-squared test or Fisher’s exact test for categorical variables as appropriate. Statistical significance was set at *P* < 0.05.

For the analyses of time-related events, a cumulative incidence function was generated using a competing risk model with all-cause death as a competing variable.

## RESULTS

### Patients

**Figure 1:**
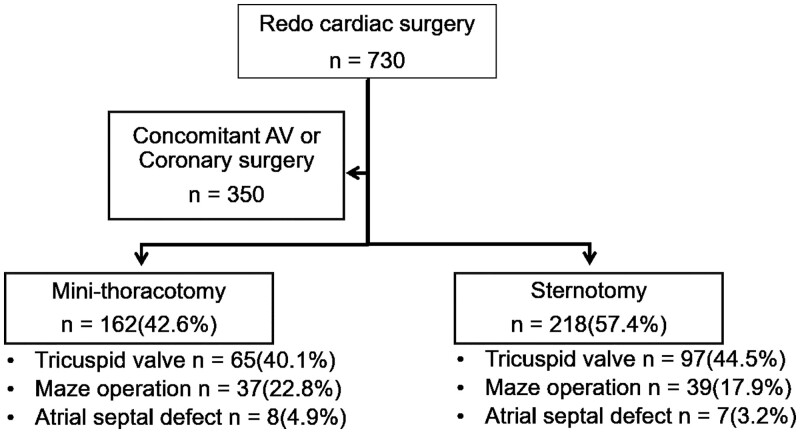
Study flow and patient characteristics. From January 2002 to July 2018, 730 consecutive adult patients underwent reoperative mitral valve surgery after a previous sternotomy at our institution. We identified 380 patients after excluding those who underwent concomitant aortic valve or coronary bypass surgeries that required a sternotomy approach. Among these, sternotomy and mini-thoracotomy approach was used in 218 (STERN group) and 162 (MINI group) patients, respectively. Concomitant procedures included tricuspid valve surgery, surgical ablation and atrial septal defect closure without significant between-group differences. AV: aortic valve.

From January 2002 to July 2018, 730 consecutive adult patients underwent redo MV surgery after a previous sternotomy at our institution. Of these, we identified 380 patients (aged 56.0 years, 228 women) after excluding those who underwent concomitant AV or coronary bypass surgeries (Fig. 1). The patients were grouped based on the surgical approaches—mini-thoracotomy (MINI group; *n* = 162) and conventional sternotomy (STERN group; *n* = 218). Patients’ baseline characteristics including echocardiographic variables are provided in Table 1. Patients in the STERN group, on average, were significantly older (55.1 [15.0] vs 51.5 [14.2] years; *P* = 0.02) and had a significantly higher prevalence of peripheral arterial occlusive disease (1.2% [2/162] vs 6.4% [14/218], *P* = 0.03) than those in the MINI group.

**Table 1:** Preoperative baseline characteristics between the mini-thoracotomy and sternotomy groups

Variable	MINI (*n* = 162)	STERN (*n* = 218)	*P*-value	SMD
Age	51.5 (14.2)	55.1 (15.0)	0.02	0.25
Female sex	103 (63.6)	125 (57.3)	0.26	0.13
Diabetes mellitus	19 (11.7)	28 (12.8)	0.87	0.03
Hypertension	35 (21.6)	63 (28.9)	0.14	0.17
Heart failure	14 (8.6)	30 (13.8)	0.17	0.16
Dyslipidaemia	23 (14.2)	22 (10.1)	0.13	0.13
PAOD	2 (1.2)	14 (6.4)	0.03	0.27
Cerebrovascular accident	17 (10.5)	32 (14.7)	0.29	0.13
COPD	5 (3.1)	10 (4.6)	0.63	0.08
Chronic kidney disease	4 (2.5)	14 (6.4)	0.12	0.19
Atrial fibrillation	81 (50.0)	120 (55.0)	0.38	0.10
Haemoglobin (g/dl)	11.8 (2.3)	11.9 (2.3)	0.58	0.06
Echocardiographic data				
LVESD (mm)	34.0 (7.5)	35.5 (8.5)	0.08	0.18
LVEDD (mm)	52.4 (8.5)	52.7 (8.3)	0.75	0.03
LA diameter (mm)	52.8 (10.8)	54.4 (11.1)	0.14	0.15
LVEF (%)	59.5 (6.9)	57.9 (9.7)	0.06	0.20
Peak TRPG, mmHg	42.4 (19.8)	41.2 (17.8)	0.55	0.06

Values are *n* (%), or mean (standard deviation), unless otherwise indicated.

COPD: chronic obstructive pulmonary disease; LA: left atrium; LVEDD: left ventricular end-diastolic dimension; LVEF: left ventricular ejection fraction; LVESD: left ventricular end-systolic dimension; PAOD: peripheral arterial occlusive disease; SMD: standardized mean difference; TRPG, tricuspid regurgitation pressure gradient.

### Operative data

Operative data are summarized in Table 2. There was no difference in previous operation types including coronary artery bypass graft (8.0% [13/162] vs 6.0% [13/218], *P* = 0.56) between the 2 groups, except MV repair, which was more frequently performed in the MINI group (43.2% [70/162] vs 31.7% [69/218], *P* = 0.03). The number of previous sternotomies did not significantly differ between the 2 groups (*P* = 0.60, Supplementary Material, Table S1). Underlying MV pathologies were not different between groups (*P* = 0.25). In 139 patients with degenerative MV disease, 31 patients had posterior leaflet prolapse (15 in MINI vs 16 in STERN), 40 had anterior leaflet prolapse (14 in MINI vs 26 in STERN), 18 had bileaflet prolapse (10 in MINI vs 8 in STERN), 35 had mitral stenosis and the pathology of 15 patients was not characterized with respect to the leaflets on echocardiography.

**Table 2:** Patients’ operative data

	MINI (*n* = 162)	STERN (*n* = 218)	*P*-value
Previous surgery			
Coronary artery bypass graft	13 (8.0)	13 (6.0)	0.56
Mitral valve replacement	57 (35.2)	95 (43.6)	0.12
Mitral valve repair	70 (43.2)	69 (31.7)	0.03
Tricuspid valve	31 (19.1)	45 (20.6)	0.82
Aortic valve replacement	19 (11.7)	30 (13.8)	0.67
Aorta	4 (2.5)	10 (4.6)	0.42
Congenital anomaly	19 (11.7)	25 (11.5)	1.00
Underlying mitral valve pathology			0.25
Degenerative	59 (36.4)	80 (36.7)	
Rheumatic	28 (17.3)	24 (11.0)	
Infective	16 (9.9)	35 (16.1)	
Congenital	6 (3.7)	9 (4.1)	
Previous prosthetic valve failure	53 (32.7)	70 (32.1)	
Mitral valve surgery			
Mitral valve replacement	129 (79.6)	197 (90.4)	<0.01
Mitral valve repair	33 (20.4)	21 (9.6)	<0.01
Concomitant procedure			
Tricuspid valve	65 (40.1)	97(44.5)	0.46
Maze operation	37 (22.8)	39 (17.9)	0.29
ASD closure	8 (4.9)	7 (3.2)	0.56
Emergency	10 (6.2)	21 (9.6)	0.30
CPB time (min)	172.6 (59.9)	183.0 (86.3)	0.19
ACC time (min)	88.3 (37.9)	99.0 (42.7)	0.01

Values are *n* (%), or mean (standard deviation), unless otherwise indicated.

ACC: aortic cross-clamp; ASD: atrial septal defect; CPB: cardiopulmonary bypass.

MV replacement was more frequently performed in the STERN group, whereas MV repair was more frequently performed in the MINI group (both *P* < 0.01). Concomitant procedures included tricuspid valve surgery in 162 (42.6%), surgical ablation in 76 (20.0%) and atrial septal defect closure in 15 (3.9%) without significant between-group differences (Fig. 1). The mean CPB time was 173 (60) min in the MINI group and 183 (86) min in the STERN group (*P* = 0.19). The mean aortic cross-clamp (ACC) time was 88 (38) min in the MINI group and 99 (43) min in the STERN group (*P* = 0.01).

The type of cardioplegic solution was described in Supplementary Material, Table S2. Histidine–tryptophan–ketoglutarate (Custodiol^®^) solution was used 87.7% [142/162] in the MINI group, whereas blood cardioplegia was used 45.0% [98/218] in the STERN group, followed by histidine–tryptophan–ketoglutarate (37.2% [81/218]).

The case numbers according to the year of surgery was provided in Supplementary Material, Table S3. The number of mini-thoracotomy has persistently increased since 2006, but the proportion rather decreased for recent 5 years comparing with that of sternotomy owing to the increase of the number of total operations.

Two patients in the MINI group required conversion to full-sternotomy because of poor visualization of the operative field in the one, and haemothorax observed in the left chest on transoesophageal echocardiography after the completion of MV surgery in the other.

Nine patients in the MINI group underwent fibrillatory arrest instead of ACC followed by cardioplegic arrest. Aortic cross-clamping was not performed in 7 patients because of severe adhesion, and in the other 2 patients owing to previous coronary bypass graft.

Two patients in the MINI group who initially attempted to undergo MV repair showed more than moderate MR on intraoperative transoesophageal echocardiography, following a second CPB to replace the valve. No patient required the second-run CPB due to post-repair significant MR in the STERN group.

One patient in the MINI group underwent wound repair of peripheral cannulation site because of lymphocele.

### Clinical outcomes

The early mortality in the MINI group was 4.3% (7/162), and risks of early major complications were as follows; LCOS, 5.6% (9/162); early stroke, 6.8% (11/162); new-onset dialysis, 6.2% (10/162); prolonged ventilation, 15.4% (25/162); and postoperative bleeding requiring exploration, 7.4% (12/162). Transfusion requirements in the MINI group were red blood cell (unit) 5.00 (3.00, 8.00), platelet (unit) 10.00 (0.00, 10.00) and fresh frozen plasma (unit) 3.00 (0.00, 5.00). The intensive care unit (ICU) stay duration (days) and hospital stay duration (days) were 2.00 (1.00, 3.00) and 8.00 (6.00, 13.00), respectively (Table 3).

**Table 3:** Early and overall clinical outcomes of the mini-thoracotomy and sternotomy groups

Outcomes	MINI (*n* = 162)	STERN (*n* = 218)
Early outcomes	No. patients (%)	
Early death	7 (4.3)	24 (11.0)
Early complications		
LCOS requiring MCS	9 (5.6)	27 (12.4)
Early stroke	11 (6.8)	31 (14.2)
Surgical bleeding	12 (7.4)	22 (10.1)
New-onset dialysis	10 (6.2)	37 (17.0)
Prolonged ventilation (>24 h)	25 (15.4)	72 (33.0)
Transfusion	Median value [interquartile range]	
Red blood cell (unit)	5.00 [3.00, 8.00]	7.00 [4.00, 11.75]
Platelet (unit)	10.00 [0.00, 10.00]	10.00 [0.00, 20.00]
Fresh frozen plasma (unit)	3.00 [0.00, 5.00]	4.00 [2.00, 8.00]
ICU stay (days)	2.00 [1.00, 3.00]	3.00 [2.00, 7.00]
Length of stay (days)	8.00 [6.00, 13.00]	14.00 [8.00, 29.00]
Overall outcomes	No. patients (%/patient-year)	
All-cause death	28 (17.3)	68 (31.2)

Values are *n* (%), or mean (standard deviation), unless otherwise indicated.

ICU: intensive care unit; LCOS: low cardiac output syndrome; MCS: mechanical cardiac support.

The early mortality in the STERN group was 11.0% (24/218), and risks of early major complications were as follows: LCOS, 12.4% (27/218); early stroke, 14.2% (31/218); new-onset dialysis, 17.0% (37/218); prolonged ventilation, 33.0% (72/218); and postoperative bleeding requiring exploration, 10.1% (22/218). Transfusion requirements in the STERN group were red blood cell (unit) 7.00 (4.00, 11.75), platelet (unit) 10.00 (0.00, 20.00) and fresh frozen plasma (unit) 4.00 (2.00, 8.00). The ICU stay duration (days) and hospital stay duration (days) were 3.00 (2.00, 7.00) and 14.00 (8.00, 29.00), respectively (Table 3).

**Figure 2:**
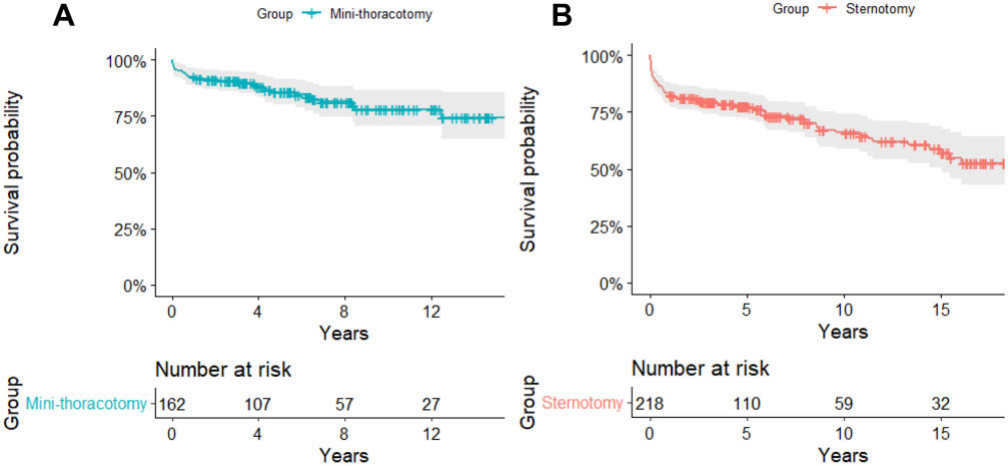
Survival curve of patients by subgroup. During the median follow-up of 68.4 (35.8–126.2) months, overall mortality occurred in 28/162 (17.3%) in the MINI group (**A**) and 68/218 (31.2%) in the STERN group (**B**).

During the median follow-up of 68.4 (35.8–126.2) months, overall mortality occurred in 96 (25.3%) patients—28/162 (17.3%) in the MINI group and 68/218 (31.2%) in the STERN group (Fig. 2). The causes of hospital and late mortalities are detailed in Supplementary Material, Table S4. MR recurrence was as follows; MV repair—7/33 (21.2%) in the MINI group and 6/21 (28.6%) in the STERN group; MV replacement—3/129 (2.3%) in the MINI group and 13/197 (6.6%) in the STERN group. Follow-up was completed for 78.9% of patients (*n* = 300).

## DISCUSSION

This study described the clinical outcomes in patients undergoing reoperative MV surgery via mini-thoracotomy and full-sternotomy approach at a single institution.

Increase in the risks related to repeat median sternotomy during reoperative cardiac surgery is well-recognized [1, 2]. The main advantage of the right mini-thoracotomy compared to repeat sternotomy is avoidance of injury to the cardiac structure associated with sternal re-entry or dissection. However, the optimal approach for MV surgery in patients with a previous sternotomy remains controversial, particularly for those who are feasible for mini-thoracotomy approach. Several studies have reported the outcomes of reoperative MV surgery with mini-thoracotomy approach. Arcidi *et al.* [6] reported the 15-year outcomes of mini-thoracotomy in patients who underwent reoperative MV surgery, and showed that the operative outcomes are comparable between mini-thoracotomy and full-sternotomy approach in these patients, with 3% early mortality. According to Romano *et al.* [5], reoperative MV surgery via mini-thoracotomy approach can be safely and effectively performed by analysing the outcomes of 450 patients. Our data were obtained from 380 patients with median follow-up of 5.7 years. A meta-analysis by Daemen *et al.* [7] has suggested that mini-thoracotomy is associated with a reduced mortality compared to sternotomy for reoperative MV surgery. However, as the authors did not account for potentially confounding factors within the studies pooled in the meta-analysis, the patients of each study may have exhibited significant differences at baseline. In this cohort, patients in the STERN group were significantly older and had a significantly higher prevalence of peripheral arterial occlusive disease than those in the MINI group.

Minimally invasive approach was known to be associated with an increased risk of stroke due to peripheral cannulation [7, 11]. The risk of early stroke in the MINI group was 6.8% in this study, which is comparable with those from previous reports [4, 7]. Kim *et al.* [8] reported the risk of early ischaemic stroke was not related to peripheral cannulation in patients undergoing AV and MV surgery.

Despite an additional incision at the right intercostal space, the rate of postoperative prolonged mechanical ventilation (>24 h) was 15.4% in the MINI group, whereas the rate in the STERN group was 33%. Arcidi *et al.* [6] demonstrated that minimally invasive MV surgery was associated with a reduced need for prolonged mechanical ventilation, and reduced hospital stay duration compared with full sternotomy. According to Romano *et al.* [5], a decreased requirement for transfusion probably indicated more stable haemodynamics, fascilitating early extubation. Moreover, shorter mechanical ventilation time would likely lead to shorter ICU and hospital stay durations and faster postoperative recovery, which was also demonstrated in our study results.

In this study cohort, 85.8% (326/380) of patients underwent MV replacement, and 32.4% (123/380) of patients underwent MV surgery for previous prosthetic valve failure. Moreover, among the rest of the patients, 13.7% (52/380) had rheumatic MV disease, and 13.4% (51/380) had infective endocarditis. According to Watkins *et al.* [9], rheumatic valve disease is more frequent in South Korea compared with that in the Western world. These factors contributed to the high MV replacement rate in this study cohort.

The previous reported that CPB time was longer with the minimally invasive approach than with the full sternotomy by 50 min [10]. However, CPB time did not significantly differ between the 2 groups in our study. Rather, ACC time was shorter in the MINI group than in the STERN group, whereas the rate of MV repair rate was higher in the MINI group (20.4% vs 9.6%, *P* < 0.01) in our study. This may be related to the better exposure of the MV by mini-thoracotomy. However, because our institute commonly performed the minimally invasive approach, the influence of each surgeon’s experience could not be ruled out. Ricci *et al.* [11] mentioned that at the beginning of their career, MV replacement was favoured over repair for cases in which complex MV repair was required.

Safe separation of the aorta seems to be a key for reoperative MV surgery via mini-thoracotomy from our institution’s experience. The foremost step is to complete the separation of the aorta to the sternum on one-lung ventilation and CPB. Upon placing the wound retractor, the separation of the aorta to the superior vena cava and the pulmonary artery can be easily achieved. In cases which separation of the aorta is difficult due to dense adhesion, a fibrillatory arrest can be performed instead of a clamped arrest. The aortic root vent cannula is placed even in fibrillatory arrest for air evacuation after the procedure.

### Limitations

The current study has several limitations. This study was a retrospective study, with inherent shortcomings. The completeness of follow-up was 78.9% in this study, which needs cautious interpretation of the results. However, in this study, as all the patient’s vital statistics was obtained from the National Population Registry of the Korea National Statistical Office, the follow-up completeness in terms of all-cause death was 100%. As our institute has a plenty of experience with the minimally invasive cardiac surgery, the results of this study may not be generalizable to those from other institutes that are less familiar with minimally invasive approaches. Also, this study included the results from 8 surgeons with a difference preference for the surgical approach. Because there was no established decision-making protocol with regard to approach, operative profiles, including each surgeon’s preferences, would be difficult to account for, leading to potential selection bias. In addition, operative techniques might have advanced throughout 16 years of the study period, and the effect of these advances may have not been sufficiently addressed. Despite the standard ICU care protocol that were applied to almost all patients in this study, potential bias in treatment decisions on patients could have not been excluded.

## CONCLUSIONS

In the setting of redo MV surgery, the mini-thoracotomy approach demonstrated acceptable postoperative outcomes and may serve as a viable alternative to conventional sternotomy when performing reoperative MV surgery.

## SUPPLEMENTARY MATERIAL

Supplementary material is available at *ICVTS* online.

**Conflict of interest:** none declared. 

### Author contributions

**Yelee Kwon:** Data curation; Formal analysis; Investigation; Methodology; Resources; Software; Visualization; Writing—original draft; Writing—review & editing. **Sung Jun Park:** Data curation; Formal analysis; Investigation; Resources; Software. **Ho Jin Kim:** Conceptualization; Formal analysis; Investigation; Methodology; Resources; Software; Supervision; Validation; Visualization; Writing—review & editing. **Joon Bum Kim:** Conceptualization; Formal analysis; Investigation; Methodology; Resources; Software; Supervision; Validation. **Sung-Ho Jung:** Conceptualization; Formal analysis; Investigation; Software; Supervision; Validation. **Suk Jung Choo:** Conceptualization; Formal analysis; Investigation; Software; Supervision; Validation. **Jae Won Lee:** Conceptualization; Investigation; Methodology; Supervision; Validation; Writing—review & editing.

### Reviewer information

Interactive CardioVascular and Thoracic Surgery thanks Francesco Patane and the other, anonymous reviewer(s) for their contribution to the peer review process of this article.

## Supplementary Material

ivab309_Supplementary_Data

## ABBREVIATIONS

ACC: Aortic cross-clamp
AV: Aortic valve
CPB: Cardiopulmonary bypass
ICU: Intensive care unit
LCOS: Low cardiac output syndrome
MR: Mitral regurgitation
MV: Mitral valve
