# Supplementary material for: Mini-thoracotomy and full-sternotomy approach for reoperative mitral valve surgery after a previous sternotomy
Source: Interact Cardiovasc Thorac Surg. 2021 Nov 22;34(3):354–60. doi: 10.1093/icvts/ivab309 (PMC8860419; doi:10.1093/icvts/ivab309)
Supplement: ivab309_Supplementary_Data [file ivab309_supplementary_data.docx]

**Supplemental Tables**

Supplemental Table E1. The number of previous sternotomy between the mini-thoracotomy and sternotomy groups

| Number of sternotomy | **MINI (n=162)** | **STERN (n=218)** | | P value=0.60 | |
| --- | --- | --- | --- | --- | --- |
| 1 | 139 (85.8) | 185 (84.9) | |  | |
| 2 | 18 (11.1) | 29 (13.3) |  | |  |
| 3 | 4 (2.5) | 4 (1.8) |  | |  |
| 4 | 1 (0.6) | 0 (0.0) |  | |  |

Values are n (%) unless otherwise indicated.

Supplemental Table E2. Type of cardioplegia between the mini-thoracotomy and sternotomy groups.

| ***Cardioplegia*** | | **MINI (n=162)** | **STERN (n=218)** | P<0.01 |
| --- | --- | --- | --- | --- |
| Blood | 8 (4.9) | 98 (45.0) |  |  |
| Del Nido | 3 (1.9) | 38 (17.4) |  |  |
| Histidine-tryptophan-ketoglutarate (HTK) | 142 (87.7) | 81 (37.2) |  |  |
| St. Thomas hospital solution No.2 | 0 | 1 (0.5) |  |  |
| Fibrillatory arrest | 9 (5.6) | 0 |  |  |

Values are n (%) unless otherwise indicated.

I6

Supplemental Table E3. The number of operations yearly

| ***Year*** | | **MINI (n=162)** | | **STERN (n=218)** | P<0.01 |
| --- | --- | --- | --- | --- | --- |
| 2002 | | 0 (0.0) | 16 (7.3) |  |  |
| 2003 | | 2 (1.2) | 13 (6.0) |  |  |
| 2004 | | 4 (2.5) | 14 (6.4) |  |  |
| 2005 | | 3 (1.9) | 17 (7.8) |  |  |
| 2006 | | 9 (5.6) | 4 (1.8) |  |  |
| 2007 | | 7 (4.3) | 6 (2.8) |  |  |
| 2008 | | 10 (6.2) | 6 (2.8) |  |  |
| 2009 | | 11 (6.8) | 6 (2.8) |  |  |
| 2010 | | 8 (4.9) | 7 ( 3.2) |  |  |
| 2011 | | 8 (4.9) | 9 ( 4.1) |  |  |
| 2012 | | 16 (9.9) | 8 ( 3.7) |  |  |
| 2013 | | 14 (8.6) | 11 ( 5.0) |  |  |
| 2014 | | 13 (8.0) | 13 ( 6.0) |  |  |
| 2015 | | 14 (8.6) | 22 (10.1) |  |  |
| 2016 | | 13 (8.0) | 19 ( 8.7) |  |  |
| 2017 | | 24 (14.8) | 32 (14.7) |  |  |
| 2018 | | 6 (3.7) | 15 ( 6.9) |  |  |

Supplemental Table E4. Cause of hospital and late mortalities between the mini-thoracotomy and sternotomy groups.

| ***Cause of death*** | | **MINI (n=162)** | **STERN (n=218)** | P=0.17 | | |
| --- | --- | --- | --- | --- | --- | --- |
| ***Hospital Mortality*** | | n=7 | n=24 |  | | |
| Low cardiac output syndrome | 2 (28.6) | 12 (50.0) |  | | |  |
| Pneumonia | 2 (28.6) | 3 (12.5) |  | | |  |
| Hypoxic brain damage | 1 (14.3) | 3 (12.5) |  | | |  |
| Hepatic failure | 2 (28.6) | 2 (8.3) |  | | |  |
| Sepsis | 0 | 2 (8.3) |  | | |  |
| Bleeding | 0 | 2 (8.3) |  | | |  |
| ***Late Mortality*** | | n=21 | n=44 |  | | |
| Pneumonia | 3 (14.3) | 2 (4.5) |  | | |  |
| Heart failure | | 1 (4.8) | 2 (4.5) | |  |  |
| Hepatic failure | | 1 (4.8) | 1 (2.3) | |  |  |
| Brain hemorrhage | | 1 (4.8) | 4 (9.1) | |  |  |
| Cancer other than heart | | 0 | 4 (9.1) | |  |  |
| Sepsis | | 2 (9.5) | 2 (4.5) | |  |  |
| Sudden cardiac death | | 0 | 2 (4.5) | |  |  |
| Unknown | | 13 (61.9) | 27 (61.4) | |  |  |

Values are n (%) unless otherwise indicated.
